# Weight-Based Framework for Predictive Modeling of Multiple Databases With Noniterative Communication Without Data Sharing: Privacy-Protecting Analytic Method for Multi-Institutional Studies

**DOI:** 10.2196/21043

**Published:** 2021-04-05

**Authors:** Ji Ae Park, Min Dong Sung, Ho Heon Kim, Yu Rang Park

**Affiliations:** 1 Department of Biomedical System Informatics Yonsei University College of Medicine Seoul Republic of Korea

**Keywords:** multi-institutional study, distributed data, data sharing, privacy-protecting methods

## Abstract

**Background:**

Securing the representativeness of study populations is crucial in biomedical research to ensure high generalizability. In this regard, using multi-institutional data have advantages in medicine. However, combining data physically is difficult as the confidential nature of biomedical data causes privacy issues. Therefore, a methodological approach is necessary when using multi-institution medical data for research to develop a model without sharing data between institutions.

**Objective:**

This study aims to develop a weight-based integrated predictive model of multi-institutional data, which does not require iterative communication between institutions, to improve average predictive performance by increasing the generalizability of the model under privacy-preserving conditions without sharing patient-level data.

**Methods:**

The weight-based integrated model generates a weight for each institutional model and builds an integrated model for multi-institutional data based on these weights. We performed 3 simulations to show the weight characteristics and to determine the number of repetitions of the weight required to obtain stable values. We also conducted an experiment using real multi-institutional data to verify the developed weight-based integrated model. We selected 10 hospitals (2845 intensive care unit [ICU] stays in total) from the electronic intensive care unit Collaborative Research Database to predict ICU mortality with 11 features. To evaluate the validity of our model, compared with a centralized model, which was developed by combining all the data of 10 hospitals, we used proportional overlap (ie, 0.5 or less indicates a significant difference at a level of .05; and 2 indicates 2 CIs overlapping completely). Standard and firth logistic regression models were applied for the 2 simulations and the experiment.

**Results:**

The results of these simulations indicate that the weight of each institution is determined by 2 factors (ie, the data size of each institution and how well each institutional model fits into the overall institutional data) and that repeatedly generating 200 weights is necessary per institution. In the experiment, the estimated area under the receiver operating characteristic curve (AUC) and 95% CIs were 81.36% (79.37%-83.36%) and 81.95% (80.03%-83.87%) in the centralized model and weight-based integrated model, respectively. The proportional overlap of the CIs for AUC in both the weight-based integrated model and the centralized model was approximately 1.70, and that of overlap of the 11 estimated odds ratios was over 1, except for 1 case.

**Conclusions:**

In the experiment where real multi-institutional data were used, our model showed similar results to the centralized model without iterative communication between institutions. In addition, our weight-based integrated model provided a weighted average model by integrating 10 models overfitted or underfitted, compared with the centralized model. The proposed weight-based integrated model is expected to provide an efficient distributed research approach as it increases the generalizability of the model and does not require iterative communication.

## Introduction

Multi-institutional studies have many advantages in that they can increase the generalizability and reproducibility of clinical results. Studies based on geographically and demographically diverse populations using multi-institutional data are increasingly common and necessary to improve generalizability [1]. This increases the applicability of study results to other settings or with other samples, as sampling bias is reduced. Sampling bias occurs when patient and disease characteristics differ from the represented patient population, and it commonly occurs in electronic health record–derived databases from single institutions, as patient populations reflect the local socioeconomic environment or specialty interests of hospitals [2].

Data accumulated in multiple institutions should be shared to realize the potential of big data in medicine. Big biomedical data networks, such as the patient-centered Scalable National Network for Effectiveness Research clinical data research network [3], Scalable Architecture for Federated Translational Inquiries Network [4], and Electronic Medical Records and Genomics (eMERGE) network [5], have been established to enable cross-institutional biomedical studies [6]. As big data are relative to volume, variety, and velocity, their serviceability depends on combining and analyzing rapidly growing data sources stored in different places via these data networks.

However, the availability of such large volumes of data is associated with privacy issues. Privacy must be protected when sensitive biomedical data are being used for research purposes, and this requires implementing several safeguards [7]. To overcome the 2 conflicting problems of privacy and data usage, a methodological solution that can analyze all partitioned data without data sharing should be considered. The current approaches toward constructing models based on multi-institution data by solving the privacy concern on patient-level data distributed across institutions can be primarily categorized into distributed computing approaches, which require iterative communication between institutions, and approaches that do not require an iterative process in terms of communication efficiency.

Among the methods that use distributed computing, federated learning has recently been proposed as a promising solution. It is a distributed computing method wherein several clients collaboratively train a shared global model with the coordination of a central server [8]. A client can be a mobile or edge device, not an institution; however, if the client is a reliable institution, it is classified as cross-silo federated learning [9]. Cross-silo federated learning aims to solve an optimization problem by setting the objective function [10] for the centralized model. In general, this optimization problem can be managed by stochastic gradient descent. Each client computes the local gradient and returns it to the server for aggregation and, accordingly, the global parameter is updated [8]. This process is repeated until the parameter converges. Various studies have also developed algorithms to establish statistical models, such as GLORE (Grid Binary LOgistic Regression) [11] for logistic regression, grid multicategory response logistic models [12] for ordinal and multinomial logistic regressions, and WebDISCO (a web service for distributed Cox model learning) [13] for the Cox model. In these studies, the global likelihood function of the centralized model was divided into local likelihood functions for each institution; to estimate the parameter maximizing the global likelihood function, the nonsensitive intermediary results were iteratively exchanged between the central server and the institutions using the Newton–Raphson method [14]. These methods can guarantee the precision of the models; however, the solutions may leak patient information owing to the disclosure of the information matrix and score vectors during iterative model learning [6].

The noniterative approach aggregates the intermediate results required for building a global model without requiring an iterative process. A typical method is meta-analysis [15], which is a conventional statistical analysis. Meta-analysis is used to estimate the effect size (eg, correlation coefficient, odds ratio [OR], and hazard ratio) of the overall institution, rather than building a predictive model. The overall effect size is estimated by averaging the effect sizes that are estimated from each institution; this method has been widely used in various studies [16-19] based on the common data model adopted by the Observational Health Data Sciences and Informatics Consortium [20]. Further, by constructing a surrogate likelihood, ODAL (one-shot distributed algorithm to perform logistic regression) [21] and ODAC (one-shot distributed algorithm for Cox model) [22] have been proposed for the logistic and Cox models, respectively; these models can estimate the global parameters in a noniterative manner without using the Newton–Raphson method. By contrast, MCCG (the multicenter collaboration gateway) [23,24], which focuses on developing a prediction model, was proposed to improve the predictive performance of a specific target institution. Rather than constructing the centralized model, this algorithm proposed a method of aggregating the models of each institution such that they are trained in a single target institution to improve the predictive performance in that target institution.

In this study, we focus on developing a noniterative algorithm that can construct predictive models from different sources without sharing horizontally partitioned data, where patient-level data are divided for the same medical information. The proposed model, referred to as the weight-based integrated model, is a predictive model reflecting the characteristics of various populations in multiple institutions without compromising privacy. We evaluated the proposed weight-based integrated model based on 2 aspects: (1) To confirm whether it provides a weighted average model with all characteristics of multi-institutional data, we evaluated its similarity with the centralized model that was developed by combining all institutional data, compared with models from different institutions, in terms of the predictive power and parameter estimation. (2) To confirm whether the proposed weight-based integrated model improves the average predictive performance by building a predictive model with generalizability, we compared the predictive power of the weight-based integrated model with that of the central model, as well as the models of each institution that were used to build weight-based integrated model, through external validation.

## Methods

### Weight-Based Integrated Model

**Figure 1:**
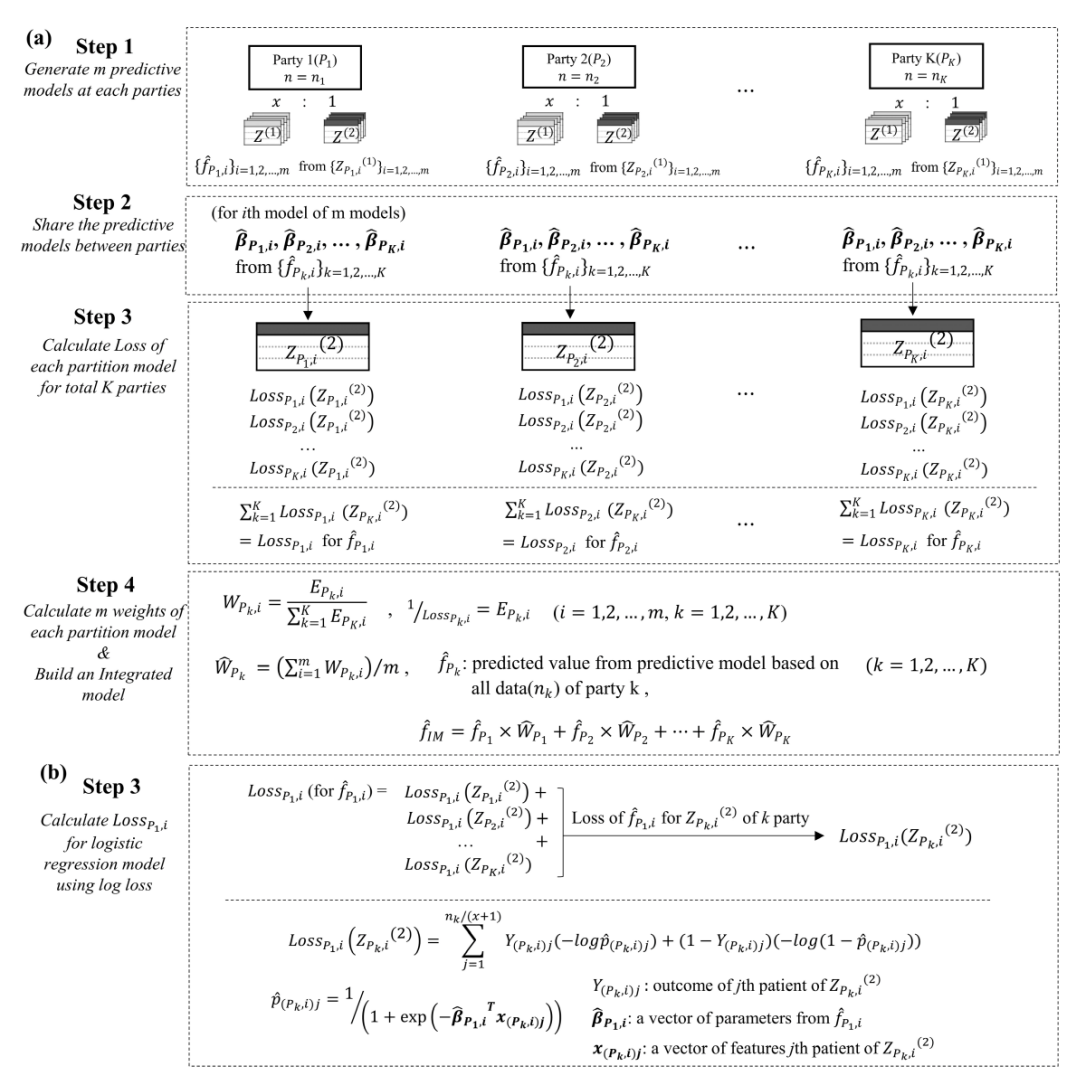
(A) Overall process of the weight-based integrated model. (B) Step 3 of the weight-based integrated model showing the process for calculating the weight using the log loss as a criterion to measure the model performance in the logistic regression model.

The proposed weight-based integrated model involves a 4-step process (Figure 1). In step 1, 2 data sets are generated by each party to estimate a predictive model and to evaluate the performance. In step 2, the parameters estimated by each party are shared between the parties. In step 3, a loss value for the model of each party is calculated by fitting the model to the data set of the entire party. The larger the loss value from the model of each party, the smaller the weight of the model. In step 4, the weight-based integrated model is constructed based on the weight of each party. To describe the 4 steps in detail, assume *K* partitioned data, each of size *n_k_*, and let *P_k_*, 1 ≤ *k* ≤ *K*, denote the *k*th partitioned data.

#### Step 1

Randomly split the *k*th party of size *n_k_* into 2 parts—the first part is *Z*^(1)^ with size (*n_k_x*)/(*x*+1), and the second part is *Z*^(2)^ with size (*n_k_*)/(*x*+1). Here, *Z*^(1)^ is used to estimate any predictive model *f*, whereas *Z*^(2)^ is used to measure the predictive performance of the estimated model *f̂* obtained from *Z*^(1)^. The data set (*Z*^(1)^, *Z*^(2)^) is generated *m* times for each *P_k_*. Let *i*, 1 ≤ *i* ≤ *m*, denote the number of data sets. 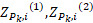 represents the *i*thdata set (*Z*^(1)^, *Z*^(2)^) of *P_k_*.

#### Step 2


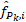
 is the *i*th model of *P_k_*_,_estimated using 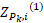, and 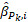 is a vector of parameters estimated from 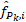. The *K* parties share *m* vectors of parameters, 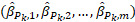, with each other.

#### Step 3

In the *k*th party, fit the *K* models, 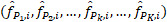, including their model, 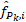, which is estimated from 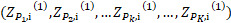 and sent from step 2 to the *i*th 
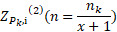
. Subsequently, calculate the loss value for each of the *K* models.


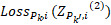
 represents loss fitting 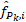 to 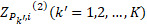. Loss for total 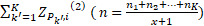 of 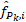 is calculated as 
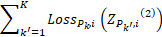
 and represents 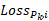. The loss function can vary depending on the model. For binary classification models (eg, the logistic regression model), the following log loss function [25], which is calculated as the negative log likelihood for probability predictions, can be used. The log loss function (or negative log likelihood function) of the logistic regression model for *N* patients is expressed as


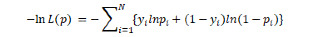


where *p_i_* = 1/(1 + exp [–*β^T^x_i_*]) is the probability of outcome of interest, *β^T^* is a vector of parameters, *x_i_* is a vector of features of the *i*th patient, and *y_i_*is a binary outcome of the *i*th patient. Figure 1B presents the process of calculating the loss for the *i*th model of party 1 (ie, 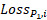) using the log loss function.

To make the weight larger as the loss becomes smaller, we define 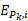 as the inverse of 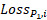, and 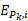 represents the goodness of fit for all *K* parties of the model of the corresponding weight.

#### Step 4

The 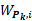, represented by *i*th weight of the partition model of *P_k_* for the integrated model, is calculated as follows:


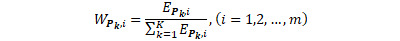


where 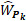 represents the final weight of the partition model based on *P_k_*, and can be obtained by averaging the 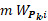. The weight-based integrated model, 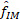, is estimated as follows, using 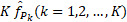, which represents a predicted value from the partition model of *P_k_* based on the total *n_k_*data. Note that 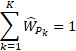.


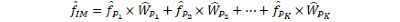


The weight calculated by the weight-based integrated model is determined by 2 factors: the data size of the party (ie, the ratio of data size to central data) and how well the model of the party fits into the data of the other parties (ie, the goodness of fit to all parties of the model from each party). In case of a party *k* with relatively large data, as the proportion of data of party *k* in the total 
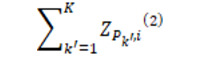
 increases, 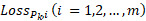 of the model of party *k* becomes small, and 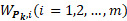 becomes larger than the other parties. In other words, a party with a large data set has a large weight, and that with a small data set has a small weight. Further, the better the model of party *k* is fitted to the data of other parties, the smaller the loss values and the greater the weights. These characteristics of weights are demonstrated in the experiments based on simulations and real data.

The parameters of the model can be also estimated based on weights from the weight-based integrated model process. In step 3, the models and weights of *K* parties are generated for every *i* repetitions. Further, the weight-based parameter can be estimated based on the *i*th weights, 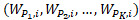, and *i*th vectors of parameters, 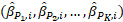, estimated from each *K* party (I = 1, 2, ..., *m*). Let 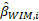 be the *i*th vector of weight-based parameters. Then, 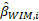 is calculated using 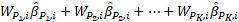; that is, parameter estimation in the weight-based integrated model is performed by calculating the weighted average of the parameters that is estimated by the models of each institution based on the weights on models of each institution. A point estimation and 95% CI estimation of a weight-based parameter can be performed using the average and (lower 2.5%, upper 97.5%) of *m* weight-based parameters, respectively.

### Simulation Study

We performed 3 simulations. The first simulation aimed to validate the optimal number of repetitions of the weight. The second and third simulations were performed to show the features of the weight calculated using the weight-based integrated model and to compare with other weighting methods. For all simulations, the standard logistic regression model was used, and 5 features were set. Three features were sampled from binomial (1, 0.5), and 2 features were sampled from normal (0, 1). The outcome was generated from the binomial (1, *p*), where 
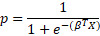
, given 5 features (*X*) and 6 parameters (*β*). We set the 6 parameters to values from –2 to 2. The values of the parameters were set to adjust the homogeneous or heterogeneous characteristics between the parties.

In the first simulation, to set an optimal *m* associated with the number of repetitions of a weight per party, we examined the change in weight by adjusting the repetition *m* under each partitioned data size *n* for the following sizes: 200, 400, 600, 800, and 1000. A total of 23 scenarios were considered, with the number of repetitions being 5 units from 5 to 50 and 50 units from 100 to 700. Three parties (A, B, and C) were considered. In this simulation, the adjustment of the homogeneous or heterogeneous characteristics of each party is not an important factor. Therefore, we generated 6 parameters for each party uniformly from [–2, 2].

The second simulation was performed to confirm the change pattern of the weights by adjusting 2 factors: the data size and the goodness of fit of the model from each party. In this simulation, we considered 2 scenarios. In the first scenario, we generated 3 parties (A, B, and C) with data sizes of 1000. One of the 3 parties was generated with a biased feature by adjusting the parameters for sampling. All 6 parameters of parties A and B were set the same. By setting 5 conditions of parameters, from parameter 1 to parameter 5, the biased degree of party C was increased as it was adjusted from parameter 1 to parameter 5. All 6 parameters of parties A and B were set equal to 1 at 5 conditions, and the parameters of party C were set to 1 at the condition of parameter 1, 0.5 at the condition of parameter 2, –0.5 at the condition of parameter 3, –1 at the condition of parameter 4, and –2 at the condition of parameter 5. That is, under the same data size, the change degree of the weights was confirmed by gradually deteriorating the goodness of fit for the entire data of the biased party C. In the second scenario, after setting one of the 3 parties to be biased, we changed the condition of data size to check the change degree of the weights according to the data size. The 6 parameters of parties A and B were set to 1, and all of party C were set to –2.

In the third simulation, we compared the weight of the weight-based integrated model with other comparable weighting methods to show the unique characteristics of the weight-based integrated model. This simulation aims to confirm to what extent the predictive performance of the integrated model using each weighting method is similar to that of the centralized model. We referred to an approach [26] of weighting strategies that investigated replicability of the performance of predictors across studies through ensembles of prediction models trained on different studies as the weights used in comparison. We chose 3 comparable weights in the approach [26] of weighting strategies: simple average (Avg), average weighted by study sample size (n-Avg), and average weighted by cross-study performance (CS-Avg). For *K* parties, with total data size N and *k*th party of size *n_k_*, Avg assigns a weight of 1/*K* to each party, and n-Avg assigns a weight of *n_k_*/*N* to each party. In addition, similar to the weight of the weight-based integrated model, CS-Avg constructs a predictive model for each party and then calculates the weight based on predictive performance for other parties. In calculating the performance of models for each party, the party used in the model is excluded. Further, the smaller the performance, the smaller the weight assigned, and the model with the lowest performance is assigned a weight of 0. An averaged value, such as the mean squared error, is used for performance measurement. For application to the logistic model of CS-Avg, we measured the performance by dividing the log loss function by the data size.

We performed 200 simulations under the same conditions. Four parties (A, B, C, and D) were constructed to build a predictive model, and another 4 validation parties were constructed to measure predictive performance. In addition, we assumed 2 scenarios, similar to the second simulation, to show the characteristics of each weight. While adjusting the data characteristics of parties under the same data sizes, and data sizes of parties under the same data characteristics, we observed the change patterns of weights and predictive performance of each weighting method. In the first scenario, the data sizes of the 4 parties were all set to 500. The 6 parameters, [*β*_0_, *β*_1_, *β*_2_, *β*_3_, *β*_4_, *β*_5_], of parties A and B were set to [0, 2, 2, 2, 2, 2], and the data characteristics of parties C and D were adjusted under the following 3 conditions: (1) 6 parameters—[0, 2, 2, 2, 2, 2], outcome generation: binomial (1, *p*); (2) 6 parameters—[0, –2, –2, 2, 2, –2], outcome generation: binomial (1, *p*); and (3) 6 parameters—[0, –2, –2, 2, 2, –2], outcome generation: binomial [1, min(0.5, *p*)]. The first condition, that is, (1), represents the same characteristics as parties A and B. By adjusting the parameter in (2) and the parameters and probability of generating an event in (3), the characteristics of parties C and D were gradually generated to be heterogeneous with parties A and B. In the second scenario, under the third condition of the first scenario, the data sizes of parties A and B were set to 500, and only the data sizes of parties C and D were changed to 500, 750, and 1000.

The data sizes of the 4 validation parties were all fixed at 500, and the data characteristics were the same as each condition of the first and the second scenarios. For example, the parameters of the 4 validation parties for condition (1) of the first scenario were set to [0, 2, 2, 2, 2, 2] in the same manner as parties A, B, C, and D. The average area under the receiver operating characteristic (ROC) curve (AUC) was measured for 4 validation parties to compare the similarity of the performance of each weighting method with that of the centralized model.

### Experiment Using Real Horizontally Partitioned Data

We used the electronic intensive care unit (eICU) Collaborative Research Database [28] to evaluate the validity of the weight model. The eICU Collaborative Research Database is a multi-institution ICU database of eICU programs across the United States, and contains approximately 200,000 admissions to ICUs monitored by 208 hospitals (data collected between 2014 and 2015).

The model to be applied to the weight-based integrated model used a logistic regression model to predict mortality after ICU admission. As features, 27 variables included in the Acute Physiology, Age, and Chronic Health Evaluation (APACHE) classification system were considered. The APACHE score is a severity-of-disease classification system [29], one of several ICU scoring systems. Therefore, we considered 27 variables from the APACHE system as mortality predictors for patients in the ICU. In the eICU database, the APACHE III score was calculated, and the 27 variables used to calculate the score were listed.

**Figure 2:**
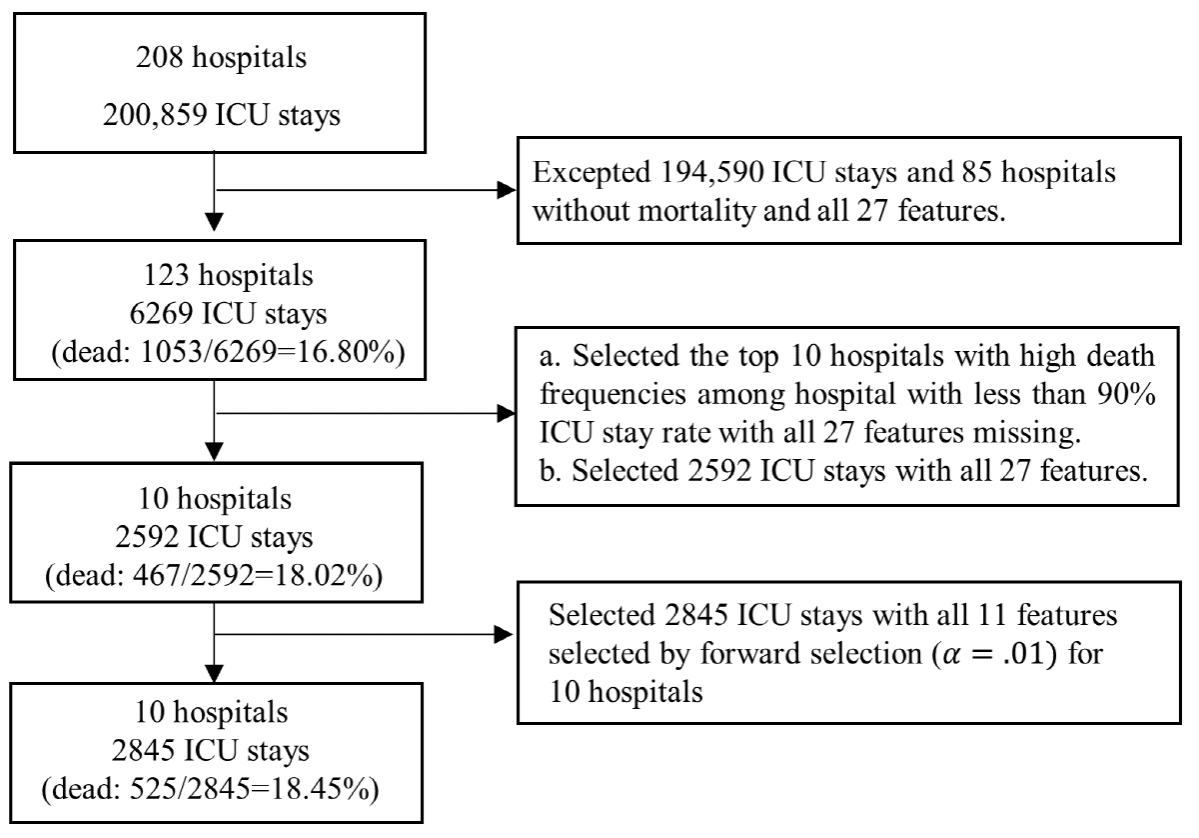
Selection process for hospitals and intensive care unit (ICU) stays.

We selected 10 hospitals with a total of 2845 ICU stays, out of 208 hospitals with a total of 200,859 ICU stays, as our horizontally partitioned data set (Figure 2). To select the horizontally partitioned data of 10 hospitals, 6269 ICU stays (123 hospitals) with both mortality and 27 feature values were selected. We selected the top 10 hospitals with higher death frequencies among those having less than 90% ICU stay rate with all 27 features missing. Moreover, 11 features were selected by forward selection (significant level: .01) of 27 features at 2592 ICU stays for 10 hospitals. The selected 11 features were Glasgow Coma Scale score, pH, blood urea nitrogen, fraction of inspired oxygen, temperature, bilirubin, albumin, age, partial pressure of carbon dioxide, partial pressure of oxygen, and pulse rate.

When developing a predictive model, the number of events compared with the number of predictors is a key factor to determine the performance of the logistic regression model [30]. The models applied to data with low events per variable produce inaccurate and biased results [31]. A total of 10 events per variable are widely used as a criterion for logistic regression models [32,33]. Most hospitals do not satisfy the 10 events per variable criterion based on the 11 features mentioned. Therefore, the firth logistic regression model [34], which can estimate unbiased parameters in data with low event frequencies, was used for accurate parameter sharing between hospitals when applying the weight-based integrated model.

### Validation and Evaluation of the Weight-Based Integrated Model

The logistic regression model was used for the simulation data, whereas the firth logistic regression model was used for the real data. To calculate the loss of 2 logistic models, we proceeded according to the process detailed in Figure 1B using the log loss function, –ln L(p). The reciprocal of the log loss risk for all data in each partition model was used as the criterion for calculating the weight. We also used the results of the first simulation as the number of repetitions required to calculate the weight. The ratio of *Z*^(1)^ to *Z*^(2)^ was 3:1 for all simulations. In addition, in real data with low event frequency, *Z*^(1)^ and *Z*^(2)^ were generated at a 1:1 ratio for both dead and alive cases to build a more stable model in *Z*^(1)^.

To evaluate the weight-based integrated model, we compared the results of the weight-based integrated model and the centralized model using 10 hospitals from the eICU database, in terms of the ROC curve, AUC, and estimated OR, on the 11 features. In addition, we used the Hosmer–Lemeshow test [35], where *P*<.05 indicates poor calibration, to assess the calibration of the proposed weight-based integrated model and centralized model for central data, along with the 10 models of each hospital.

The comparison of AUCs and ORs between the 2 models was evaluated based on the proportion of overlap of the 95% CIs. The proportion of overlap was defined as the ratio of overlap of two 95% CIs in the margin of error, which is the half-width of the 95% CI of the longer length. If a CI is remarkably short and is included in the other CI to be compared, then the proportion of overlap calculated based on the shorter CI is 2, which is a perfect match between the 2 CIs, regardless of the value of the longer CI. Therefore, the proportion of overlap was calculated based on the longer CI for a more conservative evaluation criterion. For the independent group *t* test that compares the 2 means, when the proportion of overlap is approximately 0.5 or less, it indicates that the 2-tailed *P* value is less than .05 [36]. We determined that the 2 CIs did not differ significantly at a significance level of .05 when the proportion of overlap was more than 0.5 and confirmed how close the proportion of overlap was to 2.

Based on the results of OR estimation for 11 features, we compared the results of our weight-based integrated model and conventional meta-analysis (for a fixed effect model using the inverse of the variance of the effect estimate as a weight). The meta-analysis is similar to the weight-based integrated model as the OR of a multi-institution is estimated by setting institution-specific weights and averaging the OR of each institution based on the weights, although the method of weight calculation of the meta-analysis varies from the proposed weight-based integrated model. We compared the proportional overlap of 95% CI and the relative bias of point estimates for the centralized model between the weight-based integrated model and the meta-analysis.

To perform external validation, we selected the top 5 hospitals as the external validation hospitals (ie, those with a high mortality rate and less than 90% ICU stay rate with all 27 features missing) after selecting 10 hospitals for the central data. By summarizing the AUC as a result of external validation, we confirmed whether the predictive performance on each external validation hospital in the weight-based integrated model is similar to that of the centralized model. We also evaluated whether the weight-based integrated model ultimately improves the average predictive performance when compared with a model of a single hospital through an average AUC on 5 external validations. In addition, the 3 weighting methods (ie, CS-Avg, n-Avg, and Avg) were applied to external validation and compared with the weight-based integrated model.

The simulation studies and experiments with real horizontally partitioned data were performed using R 3.6.0 (R Foundation for Statistical Computing).

## Results

### Simulation 1: Optimal Repetitions m

**Figure 3:**
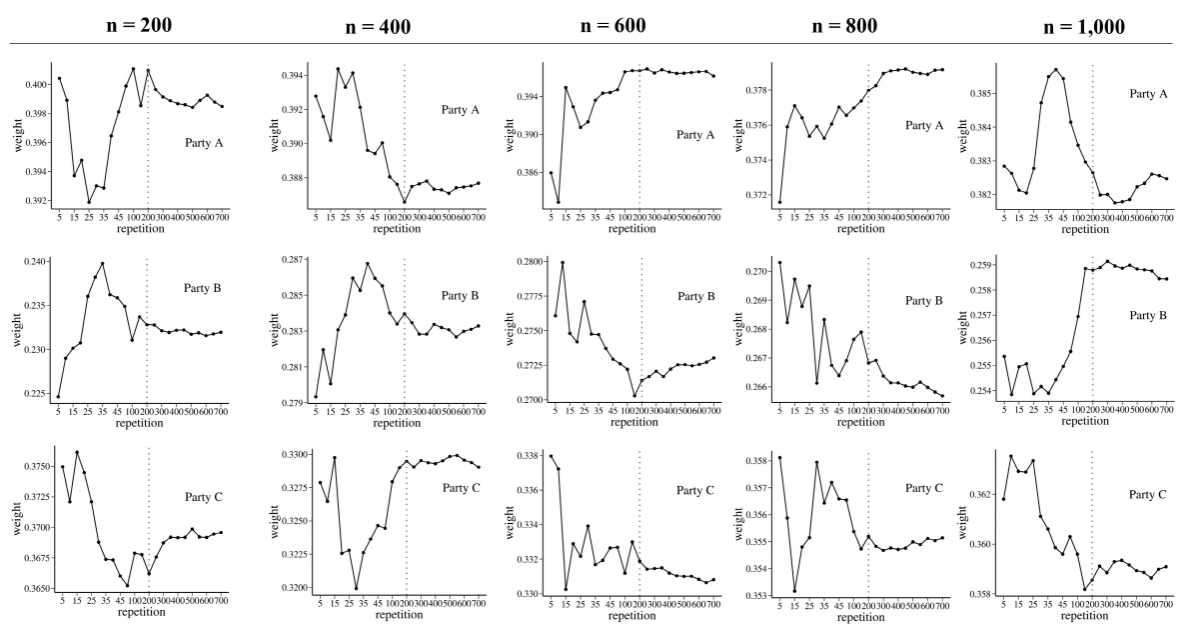
Weights of 3 parties according to the number of repetitions for sizes of 200, 400, 600, 800, and 1000. Vertical lines represent 200 repetitions.

In simulation 1, to propose optimal repetitions *m* of the weight-based integrated model, the size of each party was simulated as 200, 400, 600, 800, and 1000, and the weight values tended to stabilize as the number of repetitions increased (Figure 3). Moreover, as the data size *n* of each party decreased, the change in the weight pattern according to the number of repetitions became relatively large. For all data size *n*, graphs in Figure 3 showed a relatively flat pattern of weights after 200 repetitions. Therefore, we set *m* to 200. That is, in the second and third simulations, and the experiment using real data, we calculated the weights of each partition model and estimated the parameters of the weight-based integrated model based on 200 repetitions.

### Simulation 2: Features of the Weight Calculated From Weight-Based Integrated Model

**Figure 4:**
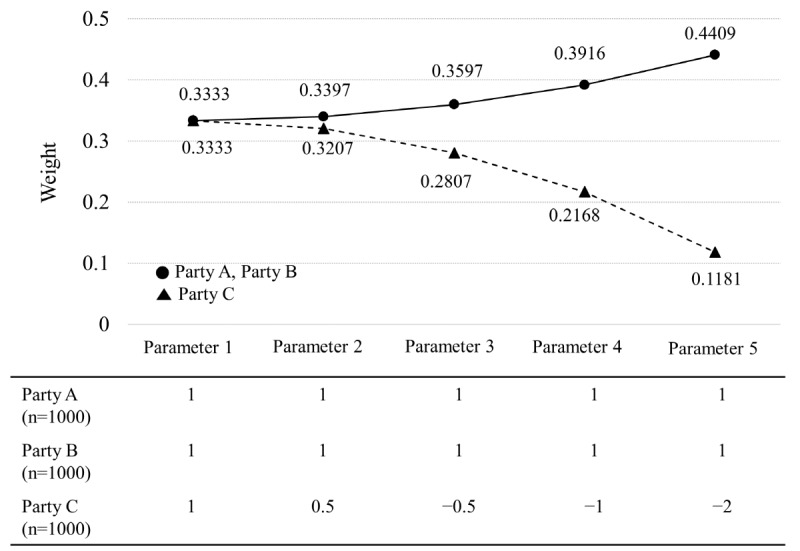
Change pattern of weights according to goodness of fit for central data (scenario 1 of simulation 2), and adjusted parameters for the 5 features of parties A, B, and C with size 1000.

To confirm the characteristics of the weights calculated using the weight-based integrated model, party C, among the 3 parties, was considered as a biased party. Figure 4 shows the results of the first scenario to confirm the change of weight according to the goodness of fit. The same weights, 0.3333, are derived for parameter 1 for all parties, where A, B, and C all have the same data. Thereafter, as the degree of bias of party C gradually increases (ie, from parameter 2 to parameter 5), the weight of party C decreases. In other words, under the same data size, the smaller the goodness of fit for the total party of a partition model with different characteristics, the smaller the weight.

**Figure 5:**
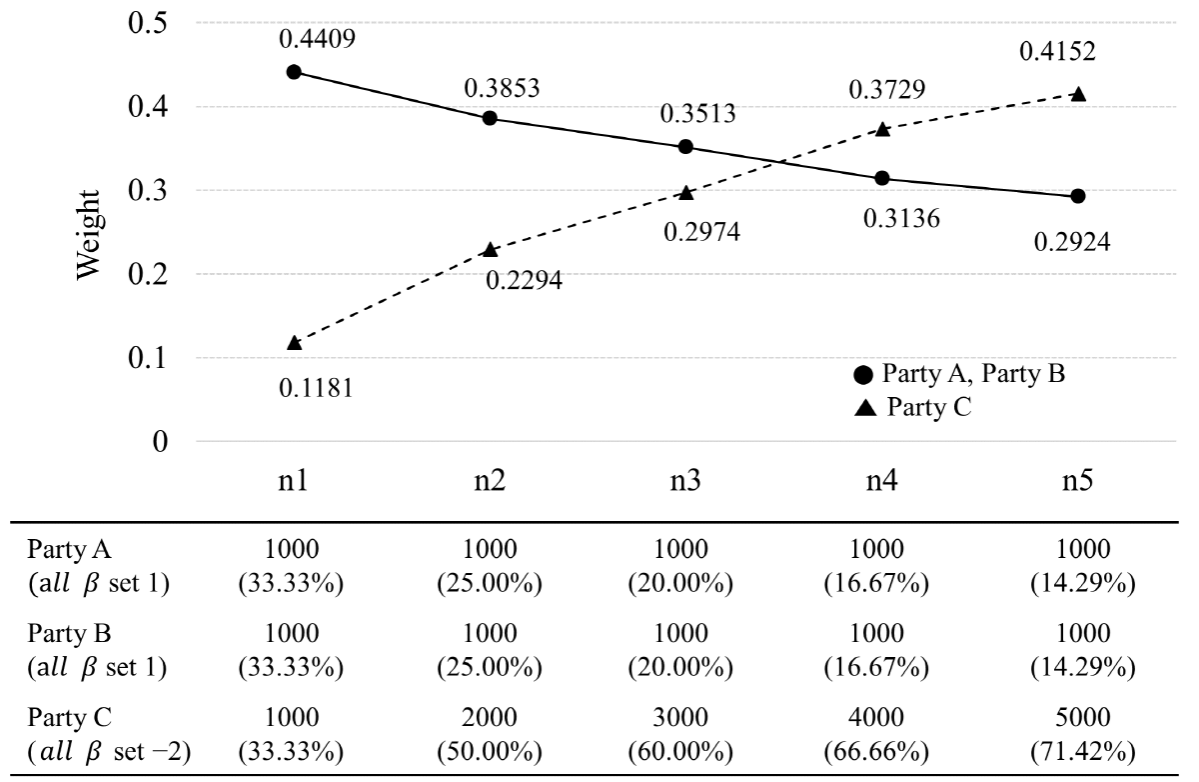
Change pattern of weights according to the ratio of data size to central data (scenario 2 of simulation 2), adjusted data sizes of party C, and ratios of data size to centralized data for parties A, B, and C.

As shown in the results of Figure 5 (scenario 2), the data size of the biased party C was gradually increased to examine the weight change according to the data size under the setting of parameter 5. When the data size of all 3 parties was equal to 1000, the weight of party C was 0.1181, which was relatively small compared with parties A and B. However, the weight of party C also increased as its data size gradually increased. In particular, after the data size of party C became 4000/6000 (66.67% of the centralized data), the weight of the biased party C became larger than that of the other 2 parties. That is, even in a biased party, the weight can be increased if the ratio of data size to the centralized data increases.

These two results of simulation 2 show that the weights of the weight-based integrated model consider not only the goodness of fit for the central data but also the ratio of data size to the central data.

### Simulation 3: Comparative Analysis With Alternative Weighting Methods

Multimedia Appendices 6 and 7 show the comparison results of 200 simulations on the weight of the weight-based integrated model and the other 3 weighting methods (CS-Avg, n-Avg, and Avg). In each simulation setting, we summarized the distribution of 200 average AUC for 4 validation parties, the difference in average AUC between each weighting method and the centralized model, and the average weights of 4 parties (A, B, C, and D), according to 200 simulations.

The results for the first scenario are shown in Multimedia Appendix 6. The data characteristics of parties C and D are gradually heterogeneous with those of party A and B as they go to the left, middle, and right. When the data sizes and characteristics of the 4 parties were all the same (the left in Multimedia Appendix 6), the distributions of the 200 average AUC of each weighting method and the centralized model were almost the same, and the average weights of parties A, B, C, and D were approximately 0.25, which is almost equal. However, as the data characteristics of parties C and D were more different from those of parties A and B (from the left to the right), the predictive performances of the 4 weighting methods were distinctly different. The distribution of the average AUC of CS-Avg showed the largest difference from that of the centralized model, and the weight-based integrated model showed the distribution of average AUC most similar to that of the centralized model. In the first scenario, as the data sizes of the 4 parties were the same, the weights of the 4 parties in both n-Avg and Avg were set equal to 0.25, and the distributions of the average AUC of both weighting methods were the same. As the data characteristics change, the weight-based integrated model and CS-Avg gradually assigned a greater weight to parties C and D. However, as CS-Avg assigned a weight of 0 to one of either A or B, the differences in weight between the 4 parties were greater than that of the weight-based integrated model.

The results for the second scenario are summarized in Multimedia Appendix 7. The data characteristics of the 4 parties were set identically with the condition corresponding to (3) of the first scenario, and the data sizes of parties C and D increased toward the left, middle, and right. Similar to the results of the first scenario, the distribution of the average AUC of the weight-based integrated model was the most similar to that of the centralized model, and the distribution of CS-Avg was the most different. As n-Avg reflects the change in data size, the distribution of average AUC differed from Avg as it goes to the right, and it was closer to the distribution of the centralized model than in the first scenario. As CS-Avg does not reflect the data size, even if the data size of parties C and D increased, the weights of the 4 parties remained almost unchanged. However, the weight-based integrated model gradually provided large weights to parties C and D with large data sizes. Furthermore, as n-Avg reflects the data size, but does not reflect the data characteristics, there was a difference from the weight of the weight-based integrated model reflecting both. Avg assigned 4 parties a fixed weight of 0.25 under any conditions.

### Validation Results on Horizontally Partitioned eICU Data

A total of 2845 ICU stays (dead: 525, alive: 2320) were arranged from 10 hospitals. Among the 2845 ICU stays, the total of *Z*^(1)^ of the entire hospital was 1430 ICU stays, and the total of *Z*^(2)^ was 1415 ICU stays (refer to Multimedia Appendix 1). Table 1 presents the results of AUC from the firth logistic regression model in each of the 10 hospitals. The predictive power of the models from each hospital differs from the smallest predictive power of 80.93% (hospital 6) to the largest predictive power of 92.00% (hospital 10).

The 200 log loss values for the total *Z*^(2)^ (n=1415) of each hospital model and the final weights of each hospital model were calculated from 200 repetitions (Table 1). A large distribution of loss in a hospital indicates that the goodness of fit of the hospital model is not good for all data from 10 hospitals. Therefore, the weight of a hospital with a relatively small loss distribution was calculated to be small. Further, a hospital with a small ratio of data size to central data (2845 ICU stays) tends to have a small weight. For example, in hospital 1, the distribution of the loss is the smallest, and the ratio of data size to the central data is the largest (510/2845, 17.93%). Therefore, the largest weight of 0.1188 was assigned to hospital 1. Conversely, hospital 10 has the largest distribution of loss, and the ratio of data size to the central data is the smallest (125/2845, 4.39%). Therefore, the smallest weight of 0.0583 was assigned to hospital 10. Hospitals 3 and 4 were given the same weight of 0.1109. However, the ratio of data size to central data in hospital 3 (268/2845, 9.42%) was smaller than that of hospital 4 (338/2845, 11.88%), and the loss distribution tended to be slightly smaller for hospital 3. As observed in the results of simulation 2, the weight of the weight-based integrated model is affected by both the ratio of the central data and the goodness of fit to the central data.

**Table 1:** AUC, log loss, and weights for 10 models of each institution (N=2845).

Hospital number	n/N (%)	AUC^a^ (95% CI)	Log loss from 200 repetitions		Weight
			Median	(Min, Max)	
1	510/2845 (17.93)	83.81% (79.99%-87.63%)	575.18	(535.45, 668.13)	0.1188
2	387/2845 (13.60)	82.14% (76.82%-87.47%)	577.40	(536.59, 754.68)	0.1181
3	268/2845 (9.42)	86.67% (81.57%-91.78%)	616.63	(547.65, 755.15)	0.1109
4	338/2845 (11.88)	86.48% (81.43%-91.53%)	617.14	(552.61, 787.62)	0.1109
5	231/2845 (8.12)	86.29% (80.19%-92.4%)	723.90	(572.31, 1814)	0.0929
6	316/2845 (11.11)	80.93% (74.02%-87.83%)	626.65	(539.71, 978.16)	0.1076
7	308/2845 (10.83)	85.95% (78.23%-93.67%)	665.89	(561.92, 1071.16)	0.1024
8	197/2845 (6.92)	83.81% (75.88%-91.73%)	712.29	(569.31, 7280.35)	0.0912
9	165/2845 (5.79)	86.63% (79.2%-94.05%)	758.66	(566.39, 1774.99)	0.0890
10	125/2845 (4.39)	92% (86.66%-97.34%)	1008.64	(634.35, 13,722.49)	0.0583

^a^AUC: area under the receiver operating characteristic curve.

The Hosmer–Lemeshow goodness-of-fit test demonstrated that the weight-based integrated model and the centralized model fit the central data well, and the 10 models of each hospital fit the data of each hospital well (all *P*>.05; Multimedia Appendix 3).

**Figure 6:**
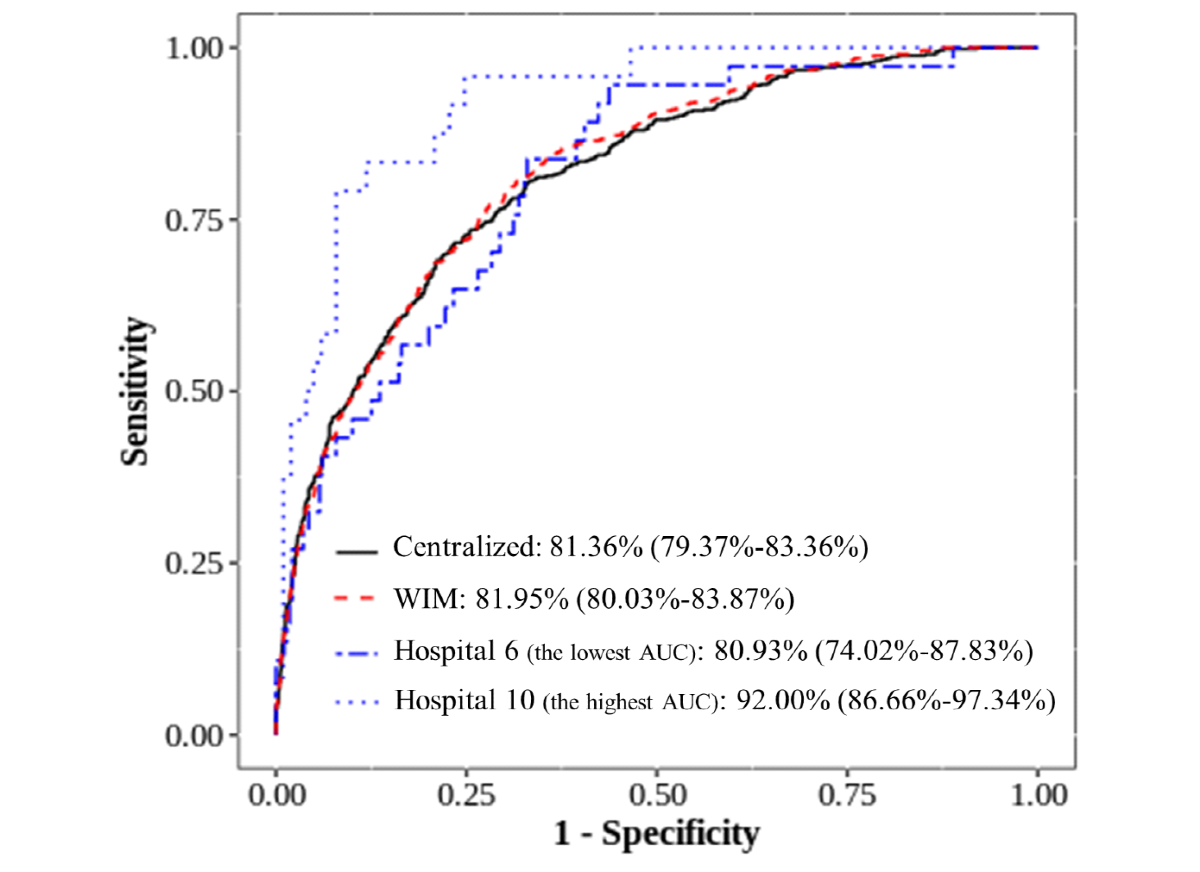
Area under the receiver operating characteristic curve (AUC), log loss from 200 repetitions, and weights. WIM: weight-based integrated model.

Figure 6 shows the ROC and AUC of the 2 models, the weight-based integrated model and the centralized model based on the central data (2845 stays), and of the 2 hospitals, hospital 6 with the lowest AUC and hospital 10 with the highest AUC (based on the data of each hospital). It was confirmed that the patterns of ROC curves for both the weight-based integrated model and the centralized model are almost the same. The estimated AUC values and 95% CIs were 81.36% (79.37%-83.36%) and 81.95% (80.03%-83.87%) in the centralized model and the weight-based integrated model, respectively (Figure 6). The proportion of overlap of CIs for AUC in both the weight-based integrated model and the centralized model was approximately 1.70. This value is much larger than 0.5, which is the level that we consider to indicate a significant difference at a significance level of .05, and is close to 2, which is the criterion for completely matching 2 CIs. Therefore, the calculated CIs for the AUC in both models were almost equal. The model of hospital 10 with the largest AUC was an overfitted model with an AUC 10% greater than for the 2 models (the weight-based integrated model and the centralized model) and the model of hospital 6 did not show much difference in the AUC value compared with the 2 models.

**Figure 7:**
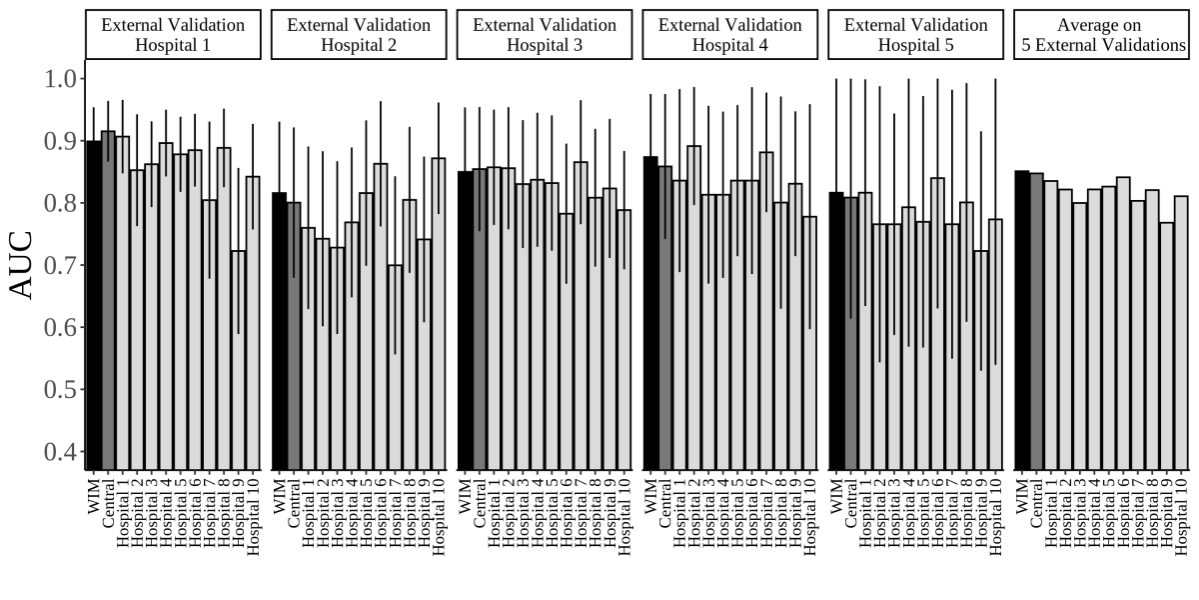
Results of AUC of external validation for the centralized model, the WIM, and 10 models of each hospital (error bar: 95% CI). Black, dark gray, and light gray indicate WIM, centralized model, and 10 models of each hospital, respectively. AUC: area under the receiver operating characteristic curve; WIM: weight-based integrated model.

A total of 535 ICU stays were selected as the 5 external validation hospitals. The frequency and rate of mortality of external validation hospitals 1, 2, 3, 4, and 5 were 20/155 (12.9%), 19/67 (28.36%), 24/226 (10.62%), 11/47 (23.4%), and 8/40 (20%), respectively. Figure 7 shows the AUC of each external validation hospital and the average AUC on 5 external validations. Multimedia Appendix 4 presents the values of the AUC (95% CI) shown in Figure 7, as well as the proportional overlap for the 95% CI of the weight-based integrated model and the centralized model. The weight-based integrated model had similar predictive performances to the centralized model in 5 external validations. In each external validation, the proportional overlap of the 95% CI for the centralized model and the weight-based integrated model was 1.59, 1.82, 1.92, 1.74, and 1.93 for external validation hospitals 1, 2, 3, 4, and 5, respectively. In addition, the average AUC was 84.74% and 85.09% for the centralized model and the weight-based integrated model, respectively. In each of the 5 external validation hospitals, a model of a single hospital out of 10 models showed higher AUC than the weight-based integrated model. However, the weight-based integrated model demonstrated the highest average predictive performance on the 5 external validation hospitals (Figure 7).

Multimedia Appendix 8 shows the comparison results of the external validation of the weight-based integrated model and 3 other weighting methods, namely, CS-Avg, n-Avg, and Avg. The proportional overlaps of the 95% CI on the AUC of the 3 weighting methods were also high, similar to those of the weight-based integrated model. In addition, the average AUCs on the 5 external validation hospitals for each weighting method were similar to each other (weight-based integrated model, 0.8509; CS-Avg, 0.8519; n-Avg, 0.8502; Avg, 0.8507).

**Figure 8:**
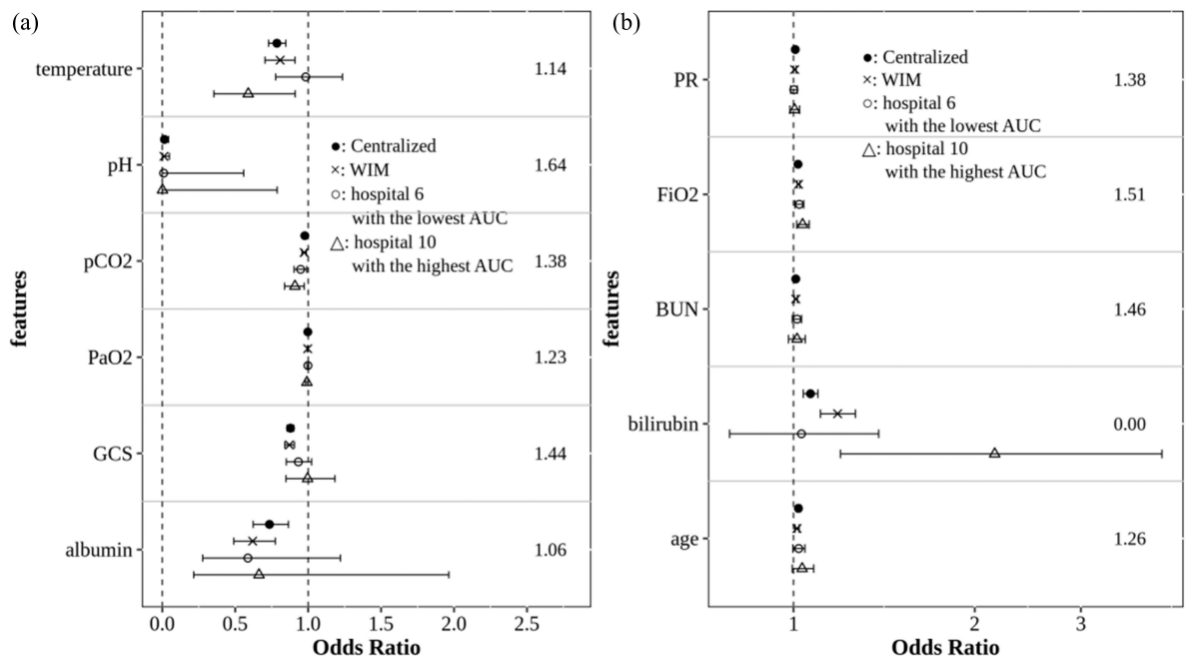
Comparison of estimated OR and 95% CI on 11 features in the firth logistic regression model: (A) features with OR < 1 and (B) features with OR > 1. The numbers on the right sides of the figures are the proportional overlap of 95% CI of OR between the WIM and the centralized model. AUC: area under the receiver operating characteristic curve; BUN: blood urea nitrogen; FiO2: fraction of inspired oxygen; GCS: Glasgow Coma Scale; OR: odds ratio; PaO2: partial pressure of oxygen; pCO2: partial pressure of carbon dioxide; PR: pulse rate; WIM: weight-based integrated model.

Figure 8 shows the OR and 95% CI of 11 features estimated using the weight-based integrated model and the centralized model, based on the central data (2845 stays), and 2 hospitals (hospital 6 with the lowest AUC and hospital 10 with the highest AUC). The 11 features were significant in both the centralized model and the weight-based integrated model, and the direction of OR significance was consistent in both models. Figure 8A presents the result of significant features with OR < 1, whereas Figure 8B presents the result of significant features with OR > 1. For the proportional overlap of 95% CI of OR between the weight-based integrated model and the centralized model, all 10 features, except bilirubin, showed a result exceeding 1 (significant difference is 0.5 at a significance level of .05), and the ORs estimated in the 2 models did not differ significantly. In bilirubin, 95% CI of the 2 models did not overlap. For each of the 11 features, ORs were estimated differently in the 10 hospitals, including hospitals 6 and 10 indicated in the graph (refer to Multimedia Appendix 2). The ORs estimated using the weight-based integrated model showed most similar estimation results to the centralized model, compared with the ORs estimated from each hospital model.

As a result of the comparison with the meta-analysis, depending on the feature, the degree of similarity to the centralized model was slightly different between the weight-based integrated model and the meta-analysis in terms of the proportional overlap of 95% CI and relative bias (Multimedia Appendix 5). Based on the criteria of the proportional overlap of 95% CI, the overlap of the weight-based integrated model and the meta-analysis for pH was 1.64 and 1.33, respectively. For Glasgow Coma Scale, pH, temperature, and partial pressure of carbon dioxide, the relative bias of the weight-based integrated model was smaller than that of the meta-analysis. These results indicate that the weight-based integrated model was closer to the centralized model than the meta-analysis. However, bilirubin, whose proportional overlap was 0 in the weight-based integrated model, showed a proportional overlap of 1.69 in the meta-analysis. In addition, the relative bias of bilirubin was 10.94% and 0.66% in the weight-based integrated model and the meta-analysis, respectively.

## Discussion

### Principal Findings

The proposed model (the weight-based integrated model) was developed to build an integrated predictive model from horizontally partitioned data without requiring physical data sharing. The weight-based integrated model is an algorithm that does not require an iterative process and can extend the model to be applied by introducing the concept of a flexible weight of a partition model. Unlike previous methodologies of building a model of central data under privacy-preserving conditions, the proposed model has the following novelties.

First, the weight-based integrated model does not require iterative communication to construct a model that approximates the centralized model. The methods that use distributed computing require an iterative exchange of information between the institutions and the central server, which is time consuming and labor intensive in practice [20]. This practical limitation can be a barrier to the application of distributed algorithms in a research consortium [20]. In cross-silo federated learning [8] with an iterative process, all clients are always available and should participate in each iteration. In other words, if a party is not available in the middle of the iteration process, the entire process is stopped. Conversely, the weight-based integrated model can build an integrated model by adjusting the weights even if a party becomes unavailable during the process. In terms of communication efficiency, naïve application of previous methodologies can yield procedures that incur exorbitant communication costs [37].

Second, the weight of the weight-based integrated model is a flexible weight derived from 2 factors, data size and the goodness of fit of each party’s model to the entire data (Figures 4 and 5). As the ratio of the data sizes of each party in the central data increases, the partition model would be closer to the centralized model. Therefore, the data size should be considered in the weighting of the partition model. If the partition model fits well to the central data, then it would be a model that describes the central data well. Therefore, the goodness of fit should also be considered with the data size. A key characteristic of the weight-based integrated model is that the weight of each partition model is derived by considering these 2 factors simultaneously. In addition, when constructing the weight-based integrated predictive model in the weight-based integrated model, the weights of the model of each party are generated *m* times (Figure 1), and the average of *m* weights is set as the final weight of the model of the party. Therefore, depending on how *m* is set, the final weights of the models of each party vary. In simulation 1, we found the optimal *m*, where the final weight remained almost unchanged while increasing the size of *m* under various data sizes of the 3 parties. The results showed that there was little change in the final weight when *m* exceeded 200 for all data sizes of the parties (Figure 3).

Third, the weight-based integrated model is a flexible algorithm in terms of scalability of the model to be applied. As the proposed model builds each partition model independently and then integrates them based on the weight, it only needs to change the form of parameters in step 2 and the loss function in step 3, depending on the model.

### Validation and Evaluation of the Weight-Based Integrated Model

We evaluated the validity of the weight-based integrated model in terms of predictive power and parameter estimation, compared with the centralized model. Experimental results using real horizontally partitioned data demonstrated that the weight-based integrated model provides a close approximation to the centralized model and improves the average predictive performance.

In terms of predictive power, the weight-based integrated model was substantially similar to the centralized model based on the results of the ROC curve and AUC. The weight-based integrated model provided a weighted average model by integrating each partition model overfitted or underfitted, compared with the centralized model (Figure 6). The multi-institutional predictive model aims to develop a generalized model that can improve the predictive performance for the data that were not used in the model. To confirm whether the proposed model satisfies this objective, we selected 5 hospitals that were not used in the weight-based integrated model and performed an external validation. Consequently, for the estimation of the AUC for each external validation hospital, the weight-based integrated model exhibited almost similar results as the centralized model. In addition, its average AUC for the 5 external validation hospitals was higher than that of the 10 models of each hospital (Figure 7, Multimedia Appendix 4).

In terms of parameter estimation, based on the results of the proportional overlap (0.5 or less indicates a significant difference at a significance level of .05; 2 indicates two CIs overlapping completely) for 95% CI of OR (Figure 8), 10 features were over 1 or 1.5. The results of parameter estimation between the weight-based integrated model and the centralized model were quite similar. However, the 95% CI of bilirubin did not overlap between the 2 models; the estimation of bilirubin was different at the significance level of 5%. As observed in the 95% CI of 10 models on each hospital for bilirubin (refer to Multimedia Appendix 2), hospital 5 with a weight of 0.0929 and hospital 10 with a weight of 0.0583 had no overlap with the centralized model. The reason that the OR for bilirubin of the weight-based integrated model differed from the centralized model is that the proportional overlap of hospital 5 with large weight was 0. Further, the estimated OR from hospital 10 was unstable and biased compared with other hospitals. The OR and 95% CI for bilirubin of the centralized model and the weight-based integrated model were 1.07 (1.04-1.10) and 1.18 (1.11-1.27), respectively (Multimedia Appendix 2). Although the 95% CI of the weight-based integrated model did not overlap with the centralized model, in the 2 models, the statistical significance of OR and the direction of interpretation are consistent, and the overall CI of the weight-based integrated model is not far off from that of the centralized model, compared with CIs of 10 hospital models.

The results of comparison with the meta-analysis in experiments using real data indicate that, for the OR estimates of 4 out of 11 features, the relative biases of the weight-based integrated model were slightly less than those of the meta-analysis. The weight-based integrated model generally showed similar results to the meta-analysis in terms of estimation of ORs. However, depending on the features, owing to the difference in weight calculation between the meta-analysis and the weight-based integrated model, there were differences in proportional overlap of 95% CI and relative bias. The weight of the meta-analysis has institution-specific characteristics. However, as it is adjusted based on the variance of an estimator of OR, the different weights are generated even for the same institution depending on which feature’s OR is estimated. By contrast, as the weights in our proposed weight-based integrated model are assigned to the model of each institution, even if the features to be estimated are different, the same weight is given to the same institution. Although the weight of the meta-analysis has feature-specific characteristics more than the weight of the weight-based integrated model, it does not represent the weight for a model of an institution unlike the weight-based integrated model. Therefore, it cannot be regarded as a weight that encompasses the purpose of building a predictive model.

When applying the weight-based integrated model, it is necessary to consider the following: To calculate the weight of each institution in the weight-based integrated model, the data of each institution is divided into *Z*^(1)^, for building the model of each institution, and *Z*^(2)^,for measuring the predictive performances of the models of all institutions. If the data size (especially the frequency of outcome of interest) of an institution is insufficient, the model of the institution generated by *Z*^(1)^ will be unstable, and it will be difficult to accurately calculate the predictive performance from *Z*^(2)^. Therefore, the data size of each institution should be sufficient to divide them into *Z*^(1)^ and *Z*^(2)^. In addition, based on the results of the external validation, the predictive performances of each of the 5 external validation hospitals were better in the model of single hospitals, compared with those of the weight-based integrated model. In other words, the weight-based integrated model may not be a good option for the purpose of improving the predictive performance of a specific hospital (of the 5 hospitals). By contrast, as the purpose for improving the average predictive performance of the 5 hospitals, the weight-based integrated model can provide a robust unified model. In our experiment using real data, the weight-based integrated model showed the best average predictive performance on 5 external validation hospitals. However, there may be cases where the weight-based integrated model does not show the best average predictive performance. For example, when a relatively heterogeneous model among the hospitals included in the weight-based integrated model exists, and the hospital exhibits heterogeneous characteristics toward all external hospitals, if the predictive performance of the model of the heterogeneous hospital in all external validation hospitals is low, the average predictive performance of the weight-based integrated model may be poor. As the weight-based integrated model averages the models of each hospital based on the weight, the overall prediction performance may be low owing to the inclusion of a heterogeneous hospital with poor predictive performance for external validation hospitals, although it is given a small weight in the weight-based integrated model. To avoid this case, it is necessary to form hospitals of the weight-based integrated model to ensure that the overall characteristics of the hospitals in which the weight-based integrated model will be applied are evenly reflected.

The weight-based integrated model is a similar algorithm to the MCCG [23,24], as it does not require an iterative communication process between institutions and constructs a generalized predictive model by integrating the models of each institution based on the weights per institution. However, the generalization process of both models varies. The weight of the weight-based integrated model is calculated by measuring the heterogeneity of the predictive performance for the central data of the models per institution in order to estimate the centralized model. Conversely, the weight of the MCCG is calculated by measuring the heterogeneity of the predictive performance for a specific target institution of the models of the source institutions used to develop the multi-institutional predictive model in order to improve the predictive performance of the target institution. Owing to this difference in the weight calculation method, the weight-based integrated model provides a generalized model by building a unified model that reflects all the characteristics of multiple institutions, whereas the MCCG provides a generalized model by changing the model through weight adjustments according to the target hospital. In the weight-based integrated model, communication occurs between institutions only once during the process of the algorithm. Conversely, the MCCG requires communication whenever the target institution changes as communication occurs between the source and target institutions. In particular, if the goal is to build a single unified predictive model to be applied to multiple institutions, the weight-based integrated model can provide a robust model. However, if the goal is to build a predictive model for a specific target institution, the MCCG can provide a better model. Therefore, an algorithm should be strategically selected according to the goal.

### Comparison With Other Weighting Methods

We demonstrated the characteristics of the weight of the weight-based integrated model through comparative analysis with other comparable weighting methods (CS-Avg, n-Avg, and Avg) [26]. The weight of the weight-based integrated model has characteristics that are calculated by considering the data size of each party and the predictive performance of central data consisting of all parties, and these characteristics were clearly distinguished from other weights, as shown in the third simulation study (Multimedia Appendix 6).

In the weight-based integrated model, the weights were adjusted as the data characteristics of the parties changed under the same data size, and the weights were adjusted as the data sizes of the parties changed under the same data characteristics. By contrast, Avg always assigned a fixed weight that does not reflect the different characteristics and data sizes of each party, and n-Avg assigned a weight that reflects only the change in the data size of each party. In addition, CS-Avg did not reflect the change in data size, but rather reflected the change in data characteristics between parties. Because CS-Avg assigns a weight of 0 to a party with the lowest performance to other parties, the party with a weight of 0 was not considered in the model. Therefore, compared with other weights, the predictive performance of CS-Avg was the most different from that of the centralized model. The weight of the weight-based integrated model distinguished from other weights reflects the characteristics of each party in the central data in terms of data size and data characteristics of each party. The weight-based integrated model with these characteristics can build a model that shows similar predictive performance as the centralized model, compared with other weighting methods.

In our experiment using real data, there were few differences in the results of external validation between the weight-based integrated model and other weighting methods as the weights assigned to the 10 hospitals differed only slightly for each weighting method (Multimedia Appendix 8). The characteristics of each weighting method were not revealed in the application of real data. However, it can be confirmed that, through a third simulation study, a difference exists in the concept from which the weight of each weighting method is derived, and the weight of the weight-based integrated model has a characteristic for estimating the centralized model.

### Limitations

It was mentioned that the weight-based integrated model is a model without an iterative process as the novelty. However, we did not evaluate its efficiency due to the absence of iterative processes in the real distributed environment. In addition, this study verified the proposed method using 2 logistic regression models, and we did not confirm the validity of the weight-based integrated model by applying other models. As shown in the results of the estimated OR for bilirubin in Figure 7, when estimating the parameters in the weight-based integrated model, inaccurate information can be provided, compared with the centralized model. As the parameters of the proposed method were estimated by assigning weights to each party’s coefficient, the parameter estimation can be influenced by the characteristics of a specific party. This limitation indicates that when a feature is estimated to be highly biased in one party, and the weight of the party is not small relative to another, it needs to interpret the estimated value carefully from the weight-based integrated model. In the future, we will explore the application and efficiency of the weight-based integrated model in a real distributed environment based on a model that has not been applied in this study.

### Conclusions

In this study, we developed a weight-based integrated model, which can build an integrated predictive model with noniterative communication between institutions. The weight-based integrated model, which uses the concept of weights for each institution, is a privacy-protecting analytic method that can reduce the burden of distributed computing and improve the average predictive performance of external validation institutions. The proposed weight-based integrated model can provide an efficient distributed research algorithm to improve the usage of multi-institutional data.

## Appendix

Multimedia Appendix 1 The frequency and rate of events for each of total, Z(1) and Z(2) in 10 hospitals.

Multimedia Appendix 2 Estimated OR in the centralized model, the weight-based integrated model, and 10 models of each hospital in experiments using real data.

Multimedia Appendix 3 Hosmer-Lemeshow goodness-of-fit tests to assess the calibration of the weight-based integrated model and centralized model for central data, and the 10 models of each hospital.

Multimedia Appendix 4 Average AUC for 5 external validation hospitals and AUC (95% CI) of each external validation hospital in the centralized model, the weight-based integrated model, and 10 models of each of the 10 hospitals.

Multimedia Appendix 5 Comparison results of the OR (95% CI) of 11 features between the weight-based integrated model and the meta-analysis.

Multimedia Appendix 6 Results of the simulation study for comparison with other weighting methods according to the change of data characteristics under the same data size.

Multimedia Appendix 7 Results of the simulation study for comparison with other weighting methods according to the change of data size under the same data characteristics.

Multimedia Appendix 8 Results of comparative analysis of external validation by the weighting methods using the eICU data.

## Abbreviations

APACHE: Acute Physiology, Age, and Chronic Health Evaluation
AUC: area under the receiver operating characteristic curve
eICU: electronic intensive care unit
GLORE: Grid Binary LOgistic Regression
ICU: intensive care unit
MCCG: multicenter collaboration gateway
OR: odds ratio
ROC: receiver operating characteristic
