# Supplementary material for: Weight-Based Framework for Predictive Modeling of Multiple Databases With Noniterative Communication Without Data Sharing: Privacy-Protecting Analytic Method for Multi-Institutional Studies
Source: JMIR Med Inform. 2021 Apr 5;9(4):e21043. doi: 10.2196/21043 (PMC8056295; doi:10.2196/21043)
Supplement: Multimedia Appendix 1 [file medinform_v9i4e21043_app1.docx]

Appendix 1. The frequency and rate of events for each of total, Z^(1)^ and Z^(2)^ in 10 hospitals.

| **Hospital  number** | **Total** | | | **Z^(1)^** | | | **Z^(2)^** | | |
| --- | --- | --- | --- | --- | --- | --- | --- | --- | --- |
|  | **n (%)** | **event, n (%)** | | **n** | **event, n (%)** | | **n(%)** | **event, n (%)** | |
| **1** | 510(17.93%) | alive | 369(72.35%) | 256 | alive | 185(72.27%) | 254(17.95%) | alive | 184(72.44%) |
|  |  | dead | 141(27.65%) |  | dead | 71(27.73%) |  | dead | 70(27.56%) |
| **2** | 387(13.6%) | alive | 311(80.36%) | 194 | alive | 156(80.41%) | 193(13.64%) | alive | 155(80.31%) |
|  |  | dead | 76(19.64%) |  | dead | 38(19.59%) |  | dead | 38(19.69%) |
| **3** | 268(9.42%) | alive | 199(74.25%) | 135 | alive | 100(74.07%) | 133(9.4%) | alive | 99(74.44%) |
|  |  | dead | 69(25.75%) |  | dead | 35(25.93%) |  | dead | 34(25.56%) |
| **4** | 338(11.88%) | alive | 287(84.91%) | 170 | alive | 144(84.71%) | 168(11.87%) | alive | 143(85.12%) |
|  |  | dead | 51(15.09%) |  | dead | 26(15.29%) |  | dead | 25(14.88%) |
| **5** | 231(8.12%) | alive | 194(83.98%) | 116 | alive | 97(83.62%) | 115(8.13%) | alive | 97(84.35%) |
|  |  | dead | 37(16.02%) |  | dead | 19(16.38%) |  | dead | 18(15.65%) |
| **6** | 316(11.11%) | alive | 279(88.29%) | 159 | alive | 140(88.05%) | 157(11.1%) | alive | 139(88.54%) |
|  |  | dead | 37(11.71%) |  | dead | 19(11.95%) |  | dead | 18(11.46%) |
| **7** | 308(10.83%) | alive | 275(89.29%) | 155 | alive | 138(89.03%) | 153(10.81%) | alive | 137(89.54%) |
|  |  | dead | 33(10.71%) |  | dead | 17(10.97%) |  | dead | 16(10.46%) |
| **8** | 197(6.92%) | alive | 168(85.28%) | 99 | alive | 84(84.85%) | 98(6.93%) | alive | 84(85.71%) |
|  |  | dead | 29(14.72%) |  | dead | 15(15.15%) |  | dead | 14(14.29%) |
| **9** | 165(5.8%) | alive | 137(83.03%) | 83 | alive | 69(83.13%) | 82(5.8%) | alive | 68(82.93%) |
|  |  | dead | 28(16.97%) |  | dead | 14(16.87%) |  | dead | 14(17.07%) |
| **10** | 125(4.39%) | alive | 101(80.8%) | 63 | alive | 51(80.95%) | 62(4.38%) | alive | 50(80.65%) |
|  |  | dead | 24(19.2%) |  | dead | 12(19.05%) |  | dead | 12(19.35%) |
| **total** | 2845 | alive | 2320(81.55%) | 1430 | alive | 1,164(81.34%) | 1415 | alive | 1415(81.75%) |
|  |  | dead | 525(18.45%) |  | dead | 266(18.59%) |  | dead | 259(18.32%) |
