# Supplementary material for: Weight-Based Framework for Predictive Modeling of Multiple Databases With Noniterative Communication Without Data Sharing: Privacy-Protecting Analytic Method for Multi-Institutional Studies
Source: JMIR Med Inform. 2021 Apr 5;9(4):e21043. doi: 10.2196/21043 (PMC8056295; doi:10.2196/21043)
Supplement: Multimedia Appendix 2 [file medinform_v9i4e21043_app2.pdf]

Appendix 2. Estimated OR in the centralized model, the weight-based integrated model, and 10 models of each hospital in experiments using real data. The numbers on the right sides of the figures are the proportion of overlap of 95% CI of OR between the centralized model and other models. WIM: weight-based integrated model.

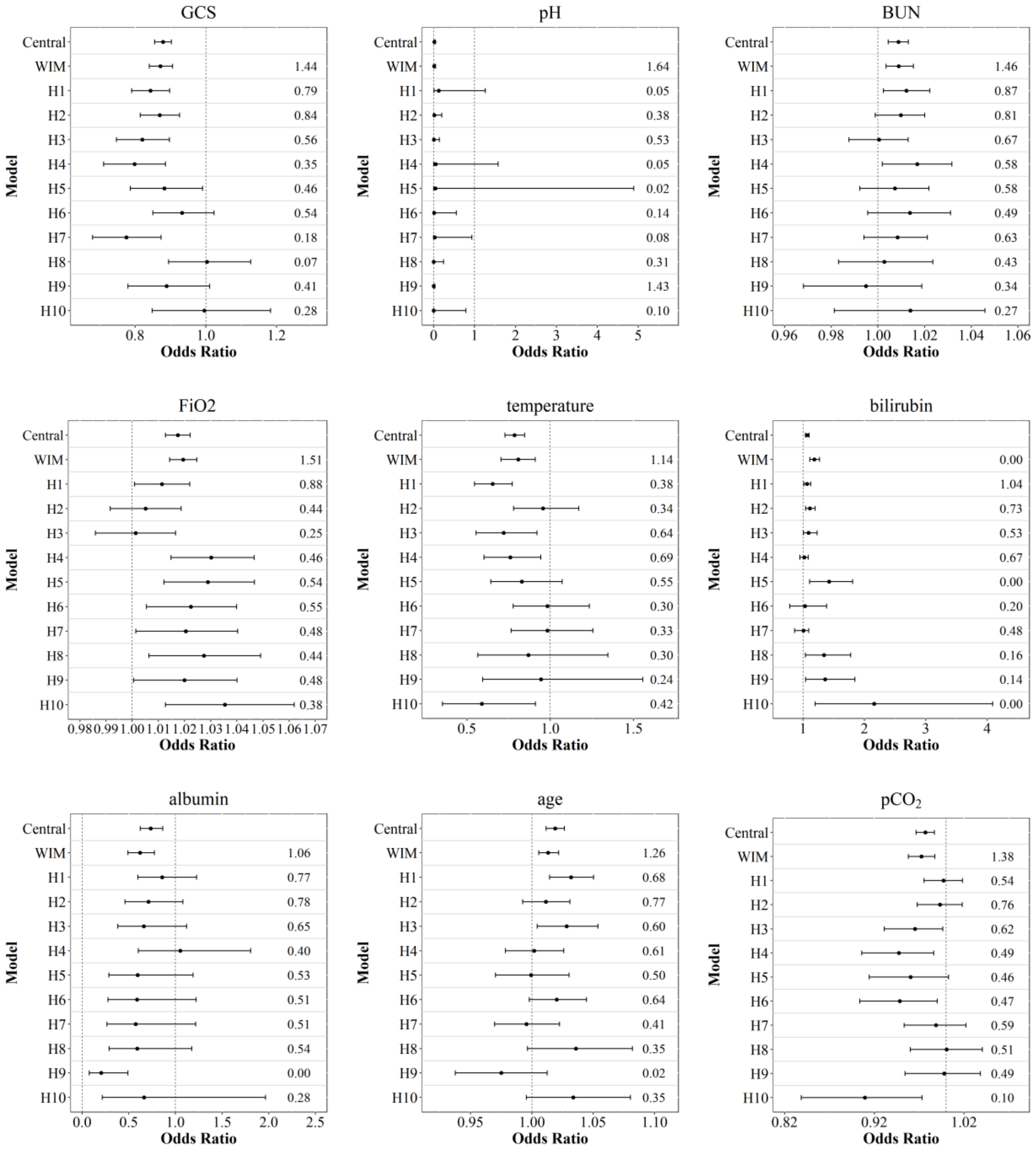

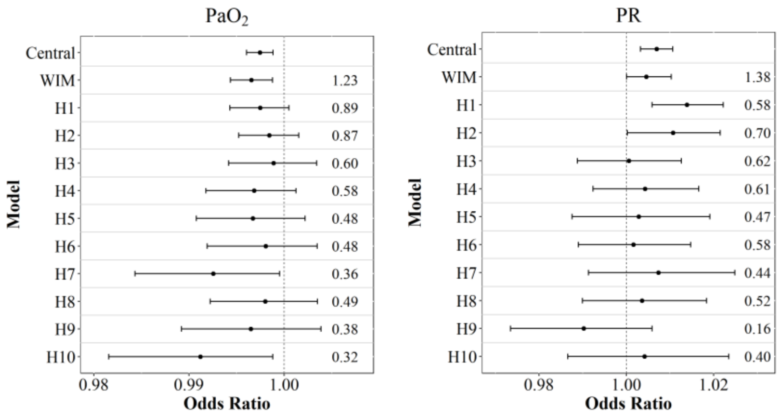

PaO_2
PR
Model
Central
WIM
H1
H2
H3
H4
H5
H6
H7
H8
H9
H10
1.23
0.89
0.87
0.60
0.58
0.48
0.48
0.36
0.49
0.38
0.32
1.38
0.58
0.70
0.62
0.61
0.47
0.58
0.44
0.52
0.16
0.40
0.98
0.99
1.00
1.02
Odds Ratio
