# Supplementary material for: Weight-Based Framework for Predictive Modeling of Multiple Databases With Noniterative Communication Without Data Sharing: Privacy-Protecting Analytic Method for Multi-Institutional Studies
Source: JMIR Med Inform. 2021 Apr 5;9(4):e21043. doi: 10.2196/21043 (PMC8056295; doi:10.2196/21043)
Supplement: Multimedia Appendix 3 [file medinform_v9i4e21043_app3.docx]

Appendix 3. Hosmer-Lemeshow goodness-of-fit tests to assess the calibration of the weight-based integrated model and centralized model for central data, and the 10 models of each hospital. WIM: weight-based integrated model.

| model | Chi-square | DF | *P* value |
| --- | --- | --- | --- |
| Central | 13.5 | 10 | 0.197 |
| WIM | 14.27 | 10 | 0.161 |
| Hospital 1 | 13.98 | 10 | 0.174 |
| Hospital 2 | 1.99 | 10 | 0.996 |
| Hospital 3 | 9.58 | 10 | 0.478 |
| Hospital 4 | 13.52 | 10 | 0.196 |
| Hospital 5 | 6.87 | 10 | 0.737 |
| Hospital 6 | 8.49 | 10 | 0.581 |
| Hospital 7 | 11.33 | 10 | 0.333 |
| Hospital 8 | 4.43 | 10 | 0.926 |
| Hospital 9 | 3.08 | 10 | 0.98 |
| Hospital 10 | 3.71 | 10 | 0.96 |
