# Supplementary material for: Weight-Based Framework for Predictive Modeling of Multiple Databases With Noniterative Communication Without Data Sharing: Privacy-Protecting Analytic Method for Multi-Institutional Studies
Source: JMIR Med Inform. 2021 Apr 5;9(4):e21043. doi: 10.2196/21043 (PMC8056295; doi:10.2196/21043)
Supplement: Multimedia Appendix 4 [file medinform_v9i4e21043_app4.docx]

Appendix 4. Average AUC for 5 external validation hospitals and AUC (95% CI) of each external validation hospital in the centralized model, the weight-based integrated model, and 10 models of each of the 10 hospitals. WIM: weight-based integrated model.

| Model | Average | External  Hospital 1 | External  Hospital 2 | External  Hospital 3 | External  Hospital 4 | External  Hospital 5 |
| --- | --- | --- | --- | --- | --- | --- |
| Hospital 1 | 0.8352 | 0.9067  (0.8475, 0.9658) | 0.7599  (0.6291, 0.8907) | 0.8573  (0.7645, 0.95) | 0.8359  (0.6886, 0.9832) | 0.8164  (0.634, 0.9988) |
| Hospital 2 | 0.8216 | 0.8526  (0.7626, 0.9426) | 0.7423 (  0.6015, 0.8831) | 0.8558  (0.7576, 0.954) | 0.8914  (0.7964, 0.9864) | 0.7656  (0.5435, 0.9878) |
| Hospital 3 | 0.7999 | 0.8622  (0.7932, 0.9312) | 0.7281  (0.589, 0.8672) | 0.8302  (0.7273, 0.9332) | 0.8131  (0.6701, 0.9562) | 0.7656  (0.5874, 0.9439) |
| Hospital 4 | 0.8217 | 0.8963  (0.8426, 0.95) | 0.7686  (0.648, 0.8893) | 0.8373  (0.7294, 0.9451) | 0.8131  (0.6793, 0.9469) | 0.793  (0.5687, 1) |
| Hospital 5 | 0.8262 | 0.8781  (0.8177, 0.9386) | 0.8158  (0.6988, 0.9328) | 0.8319  (0.7229, 0.9408) | 0.8359  (0.7143, 0.9575) | 0.7695  (0.5672, 0.9718) |
| Hospital 6 | 0.8412 | 0.8848  (0.8263, 0.9433) | 0.8629  (0.7621, 0.9638) | 0.7826  (0.6698, 0.8953) | 0.8359  (0.6855, 0.9862) | 0.8398  (0.6301, 1) |
| Hospital 7 | 0.8033 | 0.8044  (0.6781, 0.9308) | 0.6996  (0.5562, 0.8429) | 0.8655  (0.7658, 0.9653) | 0.8813  (0.7851, 0.9775) | 0.7656  (0.5493, 0.982) |
| Hospital 8 | 0.8206 | 0.8885  (0.8253, 0.9517) | 0.8048  (0.6874, 0.9223) | 0.8084  (0.6975, 0.9193) | 0.8005  (0.6298, 0.9712) | 0.8008  (0.6087, 0.9929) |
| Hospital 9 | 0.7681 | 0.7226  (0.5889, 0.8563) | 0.7412  (0.6079, 0.8746) | 0.8232  (0.7114, 0.9351) | 0.8308  (0.7143, 0.9473) | 0.7227  (0.53, 0.9153) |
| Hospital 10 | 0.8107 | 0.8422  (0.7572, 0.9272) | 0.8717  (0.7819, 0.9615) | 0.7884  (0.6931, 0.8836) | 0.7778  (0.5968, 0.9588) | 0.7734  (0.5394, 1) |
| Central | 0.8474 | 0.9152  (0.8663, 0.964) | 0.8004  (0.6794, 0.9214) | 0.8544  (0.7545, 0.9543) | 0.8586  (0.7419, 0.9752) | 0.8086  (0.6138, 1) |
| WIM | 0.8509 | 0.8989  (0.8439, 0.9539) | 0.8158  (0.7008, 0.9307) | 0.8498  (0.746, 0.9536) | 0.8737  (0.7723, 0.9752) | 0.8164  (0.6271, 1) |
| CI overlap ^a^ | | 1.59 | 1.82 | 1.92 | 1.74 | 1.93 |

^a^ proportional overlap of 95% CI of AUC between WIM and the centralized model.
