# Supplementary material for: Weight-Based Framework for Predictive Modeling of Multiple Databases With Noniterative Communication Without Data Sharing: Privacy-Protecting Analytic Method for Multi-Institutional Studies
Source: JMIR Med Inform. 2021 Apr 5;9(4):e21043. doi: 10.2196/21043 (PMC8056295; doi:10.2196/21043)
Supplement: Multimedia Appendix 5 [file medinform_v9i4e21043_app5.docx]

Appendix 5. Comparison results of the OR (95% CI) of 11 features between the weight-based integrated model and the meta-analysis. WIM: weight-based integrated model.

| Feature | Centralized model | WIM | | | Meta-analysis | | |
| --- | --- | --- | --- | --- | --- | --- | --- |
|  | OR (95% CI) | OR (95% CI) | CI overlap ^a^ | Relative bias ^b^ (%) | OR (95% CI) | CI overlap ^a^ | Relative bias ^b^ (%) |
| GCS | 0.8785  (0.8551, 0.9022) | 0.8712  (0.8398, 0.9054) | 1.44 | 0.83 | 0.8674  (0.8418, 0.8937) | 1.49 | 1.27 |
| pH | 0.0149  (0.0051, 0.0428) | 0.0115  (0.0024, 0.0484) | 1.64 | 22.33 | 0.0194  (0.0062, 0.0611) | 1.33 | 30.58 |
| BUN | 1.0087  (1.0044, 1.013) | 1.0089  (1.0034, 1.0153) | 1.46 | 0.01 | 1.0089  (1.0043, 1.0135) | 1.88 | 0.01 |
| $\mathrm{FiO}_{2}$ | 1.0174  (1.0127, 1.0221) | 1.0195  (1.0143, 1.0247) | 1.51 | 0.2 | 1.0171  (1.012, 1.0222) | 1.84 | 0.03 |
| Temperature | 0.7857  (0.7286, 0.8461) | 0.8077  (0.7042, 0.9098) | 1.14 | 2.8 | 0.8129  (0.7506, 0.8803) | 1.47 | 3.46 |
| Bilirubin | 1.067  (1.0384, 1.0978) | 1.1838  (1.1087, 1.2669) | 0 | 10.94 | 1.0741  (1.041, 1.1082) | 1.69 | 0.66 |
| Albumin | 0.7346  (0.6232, 0.8646) | 0.6194  (0.4901, 0.7747) | 1.06 | 15.69 | 0.6993  (0.5815, 0.8409) | 1.68 | 4.82 |
| Age | 1.019  (1.0117, 1.0266) | 1.0132  (1.0056, 1.0219) | 1.26 | 0.57 | 1.0153  (1.0073, 1.0234) | 1.45 | 0.37 |
| $\mathrm{pCO}_{2}$ | 0.9768  (0.9665, 0.9869) | 0.9725  (0.958, 0.9875) | 1.38 | 0.44 | 0.982  (0.9713, 0.9928) | 1.45 | 0.53 |
| $\mathrm{PaO}_{2}$ | 0.9975  (0.9961, 0.9988) | 0.9965  (0.9944, 0.9988) | 1.23 | 0.09 | 0.9974  (0.9959, 0.9989) | 1.83 | 0.01 |
| PR | 1.0069  (1.0032, 1.0106) | 1.0045  (1, 1.0103) | 1.38 | 0.23 | 1.006  (1.002, 1.01) | 1.7 | 0.09 |

^a^ proportional overlap of 95% CI of OR for the centralized model.

^b^ relative bias of point estimates of OR for the centralized model
