# Supplementary material for: Weight-Based Framework for Predictive Modeling of Multiple Databases With Noniterative Communication Without Data Sharing: Privacy-Protecting Analytic Method for Multi-Institutional Studies
Source: JMIR Med Inform. 2021 Apr 5;9(4):e21043. doi: 10.2196/21043 (PMC8056295; doi:10.2196/21043)

Appendix 6. Results of the simulation study for comparison with other weighting methods according to the change of data characteristics under the same data size. The difference in average AUC represents difference between each weighting method and centralized model on average AUC. The average weight represents an average on weights of 200 simulations. The red lines indicate the median on average AUC of centralized model and the difference in average AUC of 0. The black lines indicate the median on average AUC and the difference in average AUC, of the weight-based integrated model. WIM: weight-based integrated model.


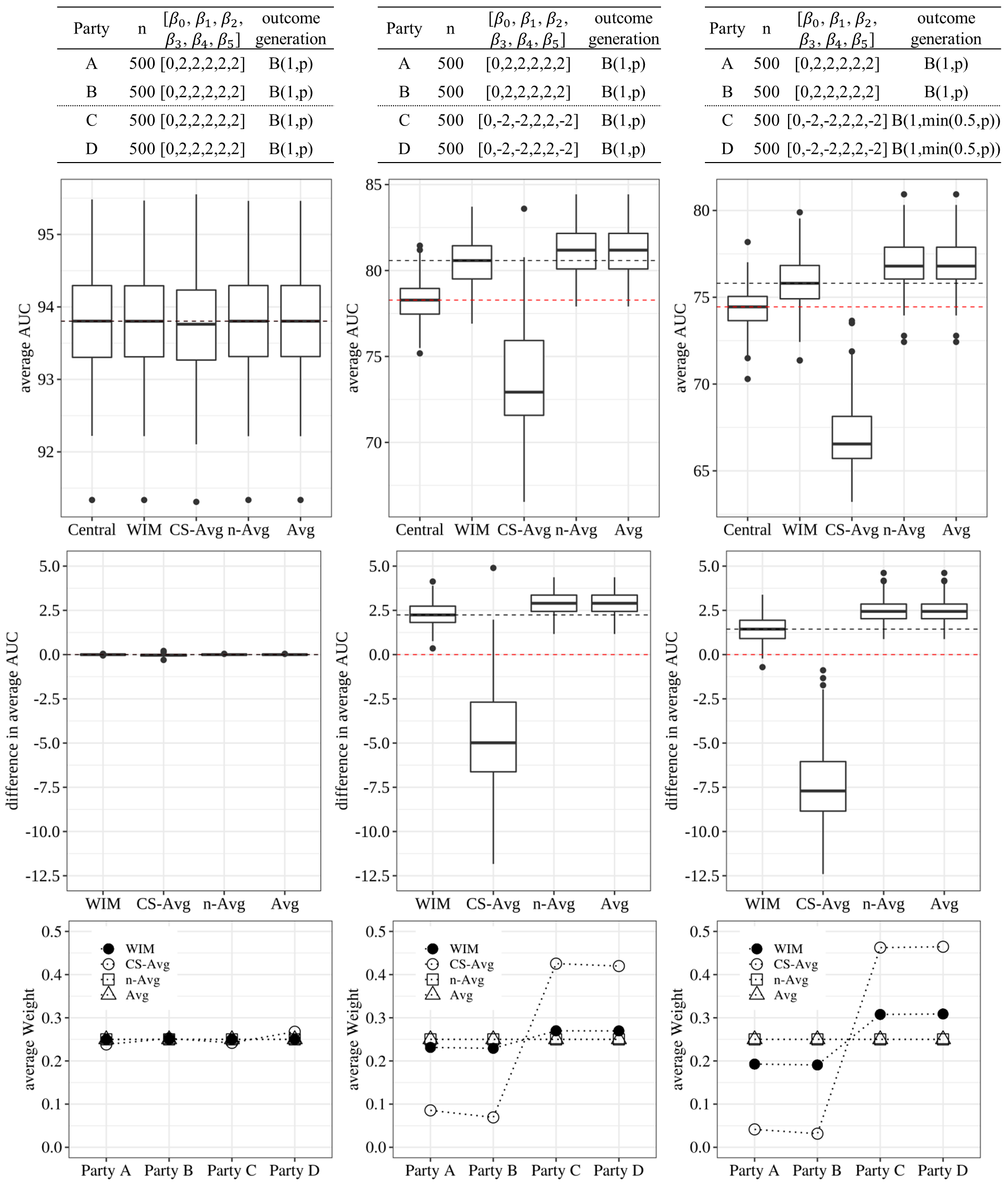

Supplement: Multimedia Appendix 6 [file medinform_v9i4e21043_app6.docx]
