# Supplementary material for: Weight-Based Framework for Predictive Modeling of Multiple Databases With Noniterative Communication Without Data Sharing: Privacy-Protecting Analytic Method for Multi-Institutional Studies
Source: JMIR Med Inform. 2021 Apr 5;9(4):e21043. doi: 10.2196/21043 (PMC8056295; doi:10.2196/21043)
Supplement: Multimedia Appendix 8 [file medinform_v9i4e21043_app8.docx]

Appendix 8. Results of comparative analysis of external validation by the weighting methods using the eICU data. WIM: weight-based integrated model.

| **Average AUC and AUC (95% CI) of each external validation hospital** | | | | | | |
| --- | --- | --- | --- | --- | --- | --- |
| Model | Average | External  Hospital 1 | External  Hospital 2 | External  Hospital 3 | External  Hospital 4 | External  Hospital 5 |
| Central | 0.8474 | 0.9152  (0.8663, 0.964) | 0.8004  (0.6794, 0.9214) | 0.8544  (0.7545, 0.9543) | 0.8586  (0.7419, 0.9752) | 0.8086  (0.6138, 1) |
| WIM | 0.8509 | 0.8989  (0.8439, 0.9539) | 0.8158  (0.7008, 0.9307) | 0.8498  (0.746, 0.9536) | 0.8737  (0.7723, 0.9752) | 0.8164  (0.6271, 1) |
|  | CI overlap ^a^ | 1.59 | 1.82 | 1.92 | 1.74 | 1.93 |
| CS-Avg | 0.8519 | 0.8981 (0.8423,  0.954) | 0.8081 (0.6919,  0.9244) | 0.8529 (0.7497,  0.9561) | 0.8838 (0.7865,  0.9812) | 0.8164 (0.6282,1) |
|  | CI overlap ^a^ | 1.57 | 1.9 | 1.94 | 1.62 | 1.93 |
| n-Avg | 0.8502 | 0.9011 (0.8446,  0.9576) | 0.8048 (0.6873,  0.9223) | 0.855  (0.7532,  0.9567) | 0.8737 (0.7714,  0.9761) | 0.8164 (0.6286,1) |
|  | CI overlap ^a^ | 1.62 | 1.93 | 1.96 | 1.75 | 1.92 |
| Avg | 0.8507 | 0.8981 (0.8425, 0.9538) | 0.8268 (0.7142, 0.9393) | 0.8459 (0.7399, 0.9519) | 0.8662 (0.7607, 0.9717) | 0.8164 (0.6307,1) |
|  | CI overlap ^a^ | 1.57 | 1.71 | 1.86 | 1.81 | 1.91 |
| **Weight of 10 hospitals** | | | | | | |
| Model | | Hospital 1 | Hospital 2 | Hospital 3 | Hospital 4 | Hospital 5 |
| WIM | | 0.1188 | 0.1181 | 0.1109 | 0.1109 | 0.0929 |
| CS-Avg | | 0.123 | 0.1252 | 0.1163 | 0.1096 | 0.1016 |
| n-Avg | | 0.1793 | 0.136 | 0.0942 | 0.1188 | 0.0812 |
| Avg | | 0.1 | 0.1 | 0.1 | 0.1 | 0.1 |
| Model | | Hospital 6 | Hospital 7 | Hospital 8 | Hospital 9 | Hospital 10 |
| WIM | | 0.1076 | 0.1024 | 0.0912 | 0.089 | 0.0583 |
| CS-Avg | | 0.1213 | 0.1041 | 0.1096 | 0.0892 | 0 |
| n-Avg | | 0.1111 | 0.1083 | 0.0692 | 0.058 | 0.0439 |
| Avg | | 0.1 | 0.1 | 0.1 | 0.1 | 0.1 |

^a^ proportional overlap of 95% CI of AUC between the weighting methods and the centralized model.
